# High-Fiber Diet or Combined With Acarbose Alleviates Heterogeneous Phenotypes of Polycystic Ovary Syndrome by Regulating Gut Microbiota

**DOI:** 10.3389/fendo.2021.806331

**Published:** 2022-02-02

**Authors:** Xuejiao Wang, Ting Xu, Rui Liu, Guojun Wu, Liping Gu, Yahui Zhang, Feng Zhang, Huaqing Fu, Yunxia Ling, Xiaohui Wei, Yunchen Luo, Jian Shen, Liping Zhao, Yongde Peng, Chenhong Zhang, Xiaoying Ding

**Affiliations:** ^1^ Department of Endocrinology and Metabolism, Shanghai General Hospital, Shanghai Jiao Tong University School of Medicine, Shanghai, China; ^2^ State Key Laboratory of Microbial Metabolism and Ministry of Education Key Laboratory of Systems Biomedicine, School of Life Sciences and Biotechnology, Shanghai Jiao Tong University, Shanghai, China; ^3^ Department of Biochemistry and Microbiology and New Jersey Institute for Food, Nutrition, and Health, School of Environmental and Biological Sciences, Rutgers University, New Brunswick, NJ, United States; ^4^ Shanghai Centre for Systems Biomedicine, Shanghai Jiao Tong University, Shanghai, China

**Keywords:** polycystic ovary syndrome, gut microbiota, clinical phenotype, dysbiosis, high-fiber diet, acarbose

## Abstract

**Objective:**

Gut microbial dysbiosis is associated with high heterogeneity of polycystic ovary syndrome (PCOS); however, studies about gut microbiota targeted clinical intervention in PCOS are limited. Our study aimed to evaluate the effects of high-fiber diet or combined with acarbose on the clinical phenotypes of PCOS, focusing on the possible influence of gut microbiota in this process.

**Methods:**

Twenty-five patients with PCOS were recruited and randomly divided into two groups, W group (n = 14) received the WTP diet (a high-fiber diet composed of whole grains, traditional Chinese medicinal foods, and prebiotics), and A group (n = 11) received the WTP diet combined with acarbose. The follow-up time was 12 weeks. The sex hormonal and glycolipid metabolic parameters, inflammatory factors, brain–gut peptides, and alteration of gut microbiota were evaluated.

**Results:**

The PCOS clinical phenotypes, inflammatory state, and brain–gut peptides secretion were all alleviated in both groups, while the hyperandrogenism, insulin resistance, and brain–gut peptides secretion were better improved in the A group. Alpha and beta diversities were altered more significantly in the A group. Amplicon sequence variants (ASVs) were clustered into 14 co-abundant groups (CAGs) as potential functional groups that may respond to the intervention. The CAGs predominantly comprised of *Bifidobacterium* and *Lactobacillus* were more enriched, while the CAGs predominantly comprised of *Bacteroides vulgatus*, *Alistipes*, *Blautia*, *Lachnospira*, and *Roseburia* were more inhibited in the A group than in W group. Moreover, the CAGs enriched in the A group had a stronger negative correlation with the luteinizing hormone (LH)/follicle-stimulating hormone (FSH) ratio, testosterone, homeostasis model assessment-insulin resistance (HOMA-IR), α-1-acid glycoprotein (α-AGP), and leptin, and positive correlation with adiponectin and spexin, while the CAGs inhibited showed an opposite trend.

**Conclusions:**

High-fiber diet could alleviate the chronic metabolic inflammation, reproductive function, and brain–gut peptides secretion of patients with PCOS, and high-fiber diet combined with acarbose could better improve the PCOS clinical phenotypes. The remodeling of gut microbiota by our intervention may play an important role in these improvements.

**Clinical Trial Registration:**

http://www.chictr.org.cn/showproj.aspx?proj=4500, ChiCTR-TRC-14005075

## Introduction

Polycystic ovary syndrome (PCOS) is a common heterogeneous endocrine-metabolic disorder in women of reproductive age. According to the 2003 Rotterdam criteria (1), PCOS is defined by the presence of the two or more of clinical manifestations: clinical or biochemical hyperandrogenism, oligo- or anovulation, and polycystic ovaries. Furthermore, PCOS often coexists with abnormal body fat distribution, insulin resistance, glycolipid metabolism disorders, and pituitary–gonad axis anomaly (2). Hyperandrogenism and insulin resistance, the two core pathogenic factors of PCOS, are positively correlated with each other (3), which suggests that PCOS exists with multiple endocrine axis dysfunctions.

European and American studies show that the consumption of cheese and high-glycemic index starchy sweets is higher and the intake of complex carbohydrates and dietary fibers is lower in patients with PCOS compared to healthy individuals (4–6). Dietary fibers intake of patients with PCOS is negatively correlated with insulin resistance, fasting insulin (FINS), glucose tolerance, and androgen levels (7). The above studies suggest that unreasonable dietary structure may result in obesity and metabolic dysfunction in patients with PCOS. Nevertheless, the mechanisms by which dysbiosis of gut microbiota can be the important trigger points of PCOS heterogeneous endocrine disorder remain largely unexplored.

A poor diet may cause the dysbiosis of gut microbiota homeostasis, termed “gut microbial dysbiosis,” and lead to obesity-related diseases (8, 9). Human studies show that patients with PCOS have gut microbial dysbiosis compared with healthy controls, and the gut microbial dysbiosis is associated with clinical parameters, including body mass index (BMI), insulin resistance, and testosterone (10–12). Furthermore, gut microbial dysbiosis may be involved in the pathogenesis of PCOS through the gut–brain axis and inflammatory reaction of host (10, 13, 14). Nowadays, there is a growing interest to use probiotics in patients with PCOS. Two studies show that the probiotics supplementation with *Lactobacillus* and *Bifidobacterium* have beneficial effects on reproductive endocrine (15) and metabolic disorders (16), respectively.

Nutritional intervention rich in dietary fibers has been gradually recognized and applied to the improvement of metabolic syndromes such as obesity and type 2 diabetes mellitus (T2DM) by selectively promoting probiotics (17, 18). Lots of studies suggest that patients with PCOS have low intake of dietary fibers (7, 19). However, studies of the dietary fiber intervention in patients with PCOS are limited. Acarbose, an α‐glucosidase inhibitor, could inhibit the absorption of starch in the small intestine and lead to starch featuring slowly digested “lente” carbohydrate and with increased delivery of starch into the large intestine eventually (20). Acarbose has been confirmed to increase the gut content of *Bifidobacteria* (21) and was once used to treat PCOS (22). We speculate that nutritional intervention with a high-fiber diet may be a feasible treatment for PCOS by remodeling the gut microbiota, and acarbose may enhance this remodeling by increasing the delivery of starch into the large intestine.

Up to now, studies on gut-microbiota-targeted intervention of PCOS are limited. This study aimed to evaluate the effects of intervention rich in dietary fibers or combined with acarbose on the clinical phenotypes of patients with PCOS and explore the possible influence of gut microbiota in this process.

## Materials and Methods

### Participants

Participants diagnosed with PCOS were recruited from the Department of Endocrinology and Metabolism and Department of Gynecology of Shanghai General Hospital (Shanghai, China). In our study, each participant met all of the three clinical manifestations based on the 2003 Rotterdam criteria, including clinical and/or biochemical hyperandrogenism, oligo- or anovulation, and polycystic ovaries (1). Exclusion criteria included the following: androgen-secreting tumors, adrenal disorders, Cushing’s syndrome, hypertension, smoking, drinking, and pregnancy. None of the participants had received treatment with hormone drugs, insulin sensitizers, anti-obesity drugs, or antibiotics within 3 months prior to enrollment.

### Study Approval

The Human Research Ethics Committee of Shanghai General Hospital (No. 2014KY091) had approved the study protocol ahead of the enrollment procedure. This clinical trial was registered in Chinese Clinical Trial Registry (ChiCTR-TRC-14005075). All participants provided written informed consent.

### Study Design

**Figure 1:**
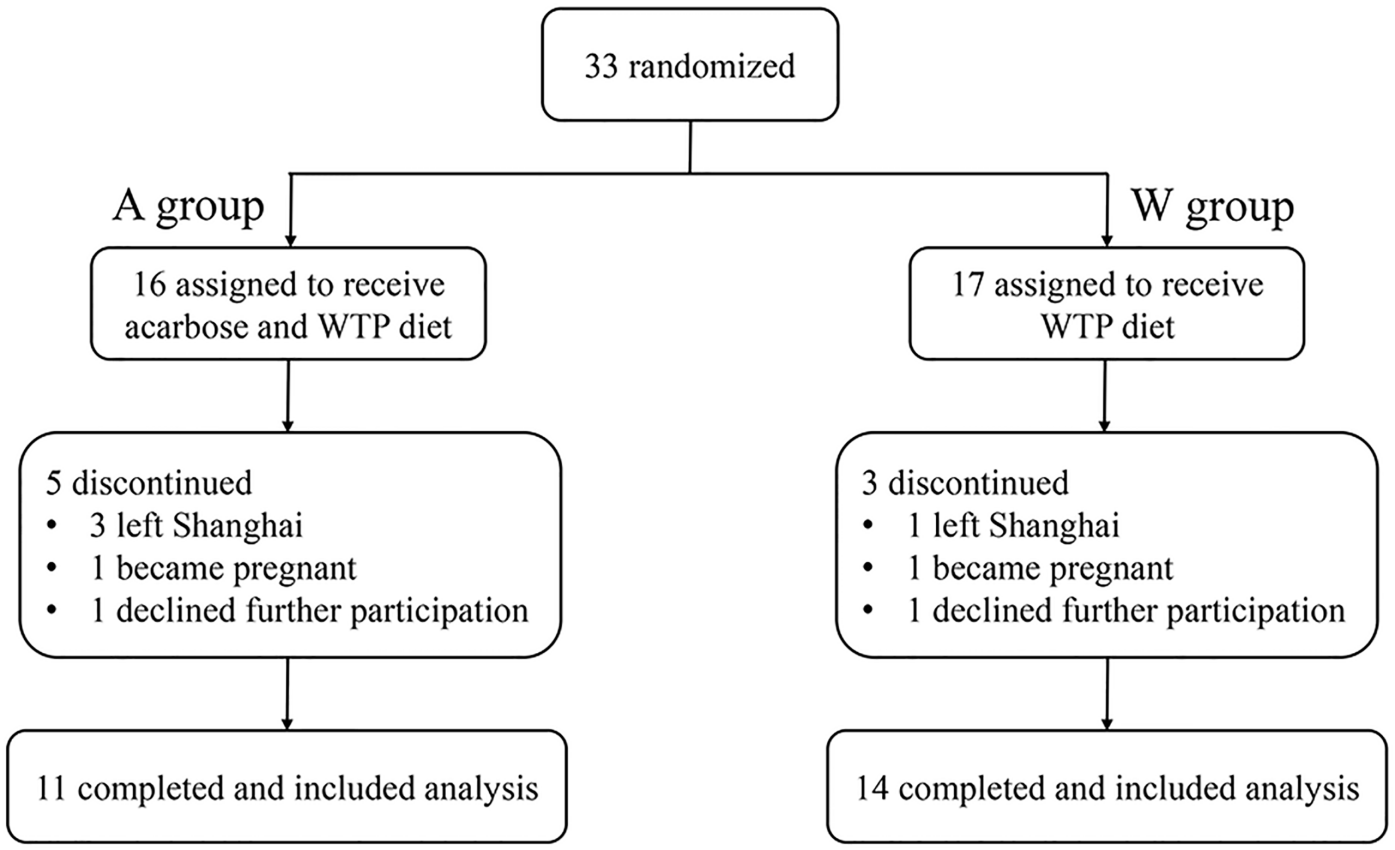
Design and participant flow of clinical trial.

This study was an open-label, randomized controlled trial. Twenty-five participants (aged 15–41 years) were randomly assigned into two groups (**Figure 1**). W group (n = 14) received a high-fiber diet composed of whole grains, traditional Chinese medicinal foods, and prebiotics (the WTP diet) for 12 weeks. A group (n = 11) received the WTP diet combined with acarbose for 12 weeks. The WTP diet included two ready-to-consume prepared foods [canned gruel and prebiotic powder; Perfect (China) Co., Zhongshan, China]. More specifically, canned gruel was a precooked mixture consisting of 11 wholegrains and traditional Chinese medicinal food that are rich in dietary fibers, including adlay, oat, white hyacinth bean, buckwheat, yam, soybean, red phaseolus bean, peanut, wolfberry, corn, and lotus seed. This canned gruel was 360 g wet weight per can prepared by the food manufacturer. Prebiotic powder was a powder preparation for infusion (20 g per bag) containing soluble dietary fibers, including resistant dextrin, maltose oligosaccharide, inulin, and fructo-oligosaccharide. All participants were instructed by a dietitian to consume at least 240 g canned gruel and 60 g prebiotic powder together with at least of 500 g fresh vegetables and with adequate amounts of meat, eggs, mushrooms, soy products fruits, and nuts per day. The detailed composition of canned gruel and prebiotic powder is shown in **Supplementary Table S2**. Participants of the A group were prescribed with acarbose (50 mg; 3 times/day) as a medication. The doses of acarbose (Glucobay, Bayer AG) were unchanged throughout the intervention. All participants received a nutrition education and physical examination at baseline.

### Outcomes

This study was conducted to explore the effects of intervention on the clinical phenotypes of PCOS and the role of gut microbiota in this process. The primary endpoints included the sex hormonal and glycolipid metabolic parameters. Gut microbiota may contribute to the development of PCOS by influencing chronic inflammation of host and gut–brain axis (23). Thus, the inflammatory factors and brain–gut peptides were also evaluated.

Food intake based on the 24-h dietary review questionnaire, anthropometric measurements, and blood and fecal samples were taken every 4 weeks (week 0, 4, 8, and 12) during the clinical intervention and follow-up. Blood samples were tested for the sex hormonal and glycolipid metabolic parameters, inflammatory factors, and brain–gut peptides. Fecal samples were used for DNA extraction and 16S rRNA gene sequencing. Dual-energy X-ray absorptiometry (DEXA) was used to measure the body composition. The Ferriman–Gallway hirsutism score was obtained at baseline and week 12.

### Laboratory Measurement

The standard 75-g oral glucose tolerance test (OGTT) was performed in all participants after an overnight fasting for at least 8 h, and blood samples were collected at the following time point (0, 30, 60, and 120 min) and stored at −80°C until analysis. Biochemical parameters were measured using an automatic biochemical analyzer (AU5800 Clinical Chemistry System, Beckman Coulter, CA, USA). Serum insulin and sex hormones were measured using an automated immunoassay system (Tosoh Bioscience AIA™—1800 Systems, TOSOH Corporation, Tokyo, Japan). HbA1c level was measured using a high-performance liquid chromatography (Bio-Rad Variant II Turbo, Bio-Rad Laboratories, München, Germany). For cytokines and brain–gut peptides, blood samples were collected according to the kit instructions. Enzyme-linked immunosorbent assays (ELISAs) were used to quantify the level of serum lipopolysaccharide (LPS)-binding protein (LBP) (Hycult Biotech, PA, USA), α-1-acid glycoprotein (α-AGP) (Assaypro Inc, MO, USA), leptin and adiponectin (DL develop, Wuhan, China), and orexin and spexin (Phoenix Pharmaceuticals Inc., Burlingame, USA). The intra- and inter-assay coefficients of variation were <5% and <10%, respectively.

### DNA Extraction and Sequencing

DNA was extracted from frozen fecal samples as we previously described (24). Forty-four samples from 11 participants in the A group (weeks 0, 4, 8, and 12) and 56 samples from 14 participants in the W group (weeks 0, 4, 8, and 12) were sequenced on the Illumina Miseq platform (Illumina, Inc., USA) with Miseq reagent kit v3 (600 cycles) (MS-102-3033, Illumina, USA). Sequencing library of the V3–V4 regions of the 16S rRNA gene was constructed based on a modified version of instruction provided by the manufacturer (Part no. 15044223 Rev. B, Illumina, USA) (25).

### Statistical Analysis

#### Clinical Data Statistical Analysis

Normal distribution of the data was calculated with the Kolmogorov–Smirnov test using SPSS statistical software 19. Variables that were not normally distributed were log-transformed before analysis. Data were expressed as means ± standard error of mean (SEM) unless otherwise stated. For the clinical and laboratory variables, one-way repeated-measures analysis of variance (ANOVA) with Dunnett’s *post-hoc* test (two tailed) was used for intra-group comparison of different time points (weeks 0, 4, 8, and 12), a paired t-test (two tailed) was used for intra-group comparison of week 12 with baseline, and a Mann–Whitney test was used to analyze differences between the A and W group at the same time point. A *p*-value <0.05 was considered to be statistically significant, *p < 0.05, **p < 0.01 and ***p < 0.001. These statistical analyses were performed using GraphPad Prism software 6.

#### Bioinformatics and Statistical Analysis

The 16S rRNA gene sequence data were processed and analyzed on the QIIME2 software (v2018.11) (26). The raw sequence data was demultiplexed and then denoised with DADA2 pipeline (q2-dada2 plugin) (27) to obtain the amplicon sequence variants (ASVs) frequency data table. Alpha diversity metrics (observed ASVs and Shannon index), beta diversity metric (Bray–Curtis and UniFrac distance), and principal coordinate analysis (PCoA) were performed using the q2-diversity after rarefying the samples to 11,000 sequences per sample. Taxonomic assignment for ASVs was performed *via* the q2-feature classifier (28) using the SILVA rRNA gene database (29). The alpha diversity indexes were compared using the analysis of ANOVA or Mann–Whitney test as the clinical indexes. The intervention-induced structural shifts of gut microbiota were evaluated using Bray–Curtis and weighted UniFrac distances, visualized by PCoA plot using GraphPad Prism software 6, and assessed by the Permutation Multivariate Analysis of Variance (PERMANOVA) analysis using R “vegan” package with 9,999 permutations.

ASVs shared by at least 20% of all samples were considered as prevalent ASVs. The correlation coefficients between the ASVs were calculated by SparCC algorithm (30). The correlations were converted to a correlation distance (1 − correlation coefficients) and then clustered into 14 co-abundant groups (CAGs) using the Ward clustering and PERMANOVA with 9,999 permutations. The ASVs were clustered using the Ward clustering algorithm *via* MATLAB, and then, the CAG network was visualized in Cytoscape. Wilcoxon matched-pairs signed-rank test was used to compare the relative abundance of CAGs on weeks 4, 8, 12 vs baseline in the same group. A Mann-Whitney test was used to compare differences between the A and W group at the same time point. *p* < 0.05 was considered as differential variable. Microbiome Multivariable Association with Linear Models 2 (MaAslin2) was used to calculate the multivariable associations *via* generalized linear regression between CAGs and clinical parameters with subjects as random effect and adjustment of age.

## Results

### Changes in Macronutrient and Dietary Fibers Intake in Patients with PCOS

At baseline, there was no significant difference in daily energy and macronutrient intakes between the two groups (**Table 1**). Both of the two groups were given the WTP diet with a large amount of diverse dietary fibers to perturb the gut ecosystem of participants for 12 weeks. After the intervention, by design, both the two groups showed a significantly higher intake of dietary fibers compared with baseline. Participants in the W group also showed a significant decrease in fat intake from week 0 to week 12. The intakes of daily energy and macronutrient had no significant difference between the two groups at week 12.

**Table 1:** Daily energy and macronutrient intake of PCOS before and during the intervention.

Daily intake	A group (n = 11)		W group (n = 14)	
	Week 0	Week 12	Week 0	Week 12
Energy, kcal	1,716.24 ± 769.21	1,380.63 ± 240.76	1,624.82 ± 480.78	1,484.82 ± 301.58
Fat, g	57.5 ± 29.33	39.48 ± 8.14	59.73 ± 20.43	45.46 ± 8.98*
Protein, g	71.32 ± 39.08	50.6 ± 17.39	66.3 ± 20.35	58.11 ± 18.92
Carbohydrate, g	243.44 ± 102.76	241.4 ± 37.01	219.86 ± 80.26	243.55 ± 51.19
Fiber, g	15.07 ± 7.8	51.93 ± 7.7***	14.35 ± 9.74	48.27 ± 8.48***
Insoluble fiber, g	9.36 ± 4.85	26.87 ± 5.16***	8.91 ± 6.05	24.82 ± 5.59***
Soluble fiber, g	5.71 ± 2.96	25.07 ± 2.73***	5.44 ± 3.69	23.45 ± 3.06***

Data are means ± SD. Paired t test (two-tailed), *p < 0.05, ***p < 0.001 vs. week 0 in the same group; unpaired t-test (two-tailed), no significant difference between the A and W group at the same time point.

### Improvement of Clinical Parameters, Inflammatory Factors, and Brain–Gut Peptides in Patients With PCOS

The clinical data of all participants (A group, n = 11; W group, n = 14) are summarized in **Supplementary Table S1**. Clinical parameters did not differ between the two groups at baseline.

**Figure 2:**
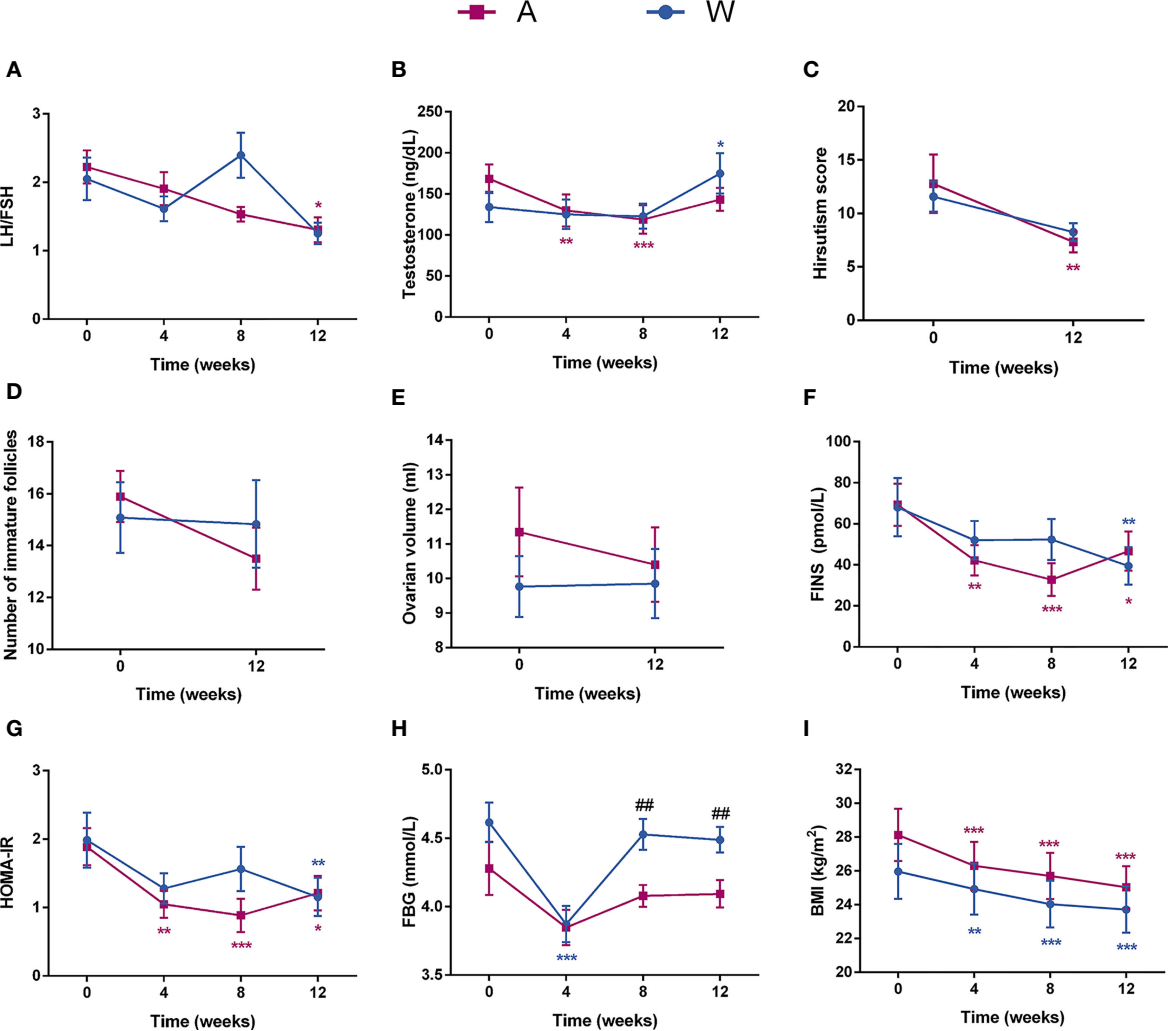
The sex hormonal and metabolic disorders were improved in patients with PCOS after the intervention. Changes in **(A)** the LH/FSH ratio, **(B)** testosterone, **(C)** hirsutism score, **(D)** number of immature follicles, **(E)** ovarian volume, **(F)** FINS, **(G)** HOMA-IR, **(H)** FBG, and **(I)** BMI for participants during the intervention are shown. Date expressed as mean ± SEM. *p < 0.05, **p < 0.01, and ***p < 0.001 for comparison with the week 0 value for the same group, red for A group and blue for W group. ^##^ *p* < 0.01 for comparison of A group with W group value at the same time point. LH, luteinizing hormone; FSH, follicular stimulating hormone; FINS, fasting plasma insulin; HOMA-IR, homeostasis model assessment for insulin resistance index; FBG, fasting blood glucose; BMI, body mass index.

The luteinizing hormone (LH)/follicle-stimulating hormone (FSH) ratio of the A group showed a steady decrease trend during the intervention, significant decrease at week 12, and significant lower level compared to the W group at week 8 (**Figure 2A**). The testosterone level of the A group significantly decreased at weeks 4 and 8, while the W group showed no significant change at this two time points and, in contrast, significantly increased at week 12 (**Figure 2B**). The hirsutism score of the A group was significantly lower after the intervention (**Figure 2C**). The result of transvaginal B-ultrasound showed that the ovarian volume and the number of immature follicles of the A group had a remarkable decrease after the intervention, while the W group had no change (**Figures 2D, E** **)**. Three participants had unplanned pregnancy during the intervention and successfully delivered afterwards; especially, one of them in the A group had multiple failures of assisted reproductive technology in the past.

The abnormal level of blood glucose, hyperinsulinemia, and insulin resistance of the two groups were improved after the intervention (**Figures 2F–H** and **Supplementary Table S1**). The A group had a better improvement of hyperinsulinemia and insulin resistance than the W group at weeks 4 and 8. After the intervention, both of the two groups showed a significant decrease in BMI (**Figure 2I**) and blood lipid levels (**Supplementary Table S1**).

**Figure 3:**
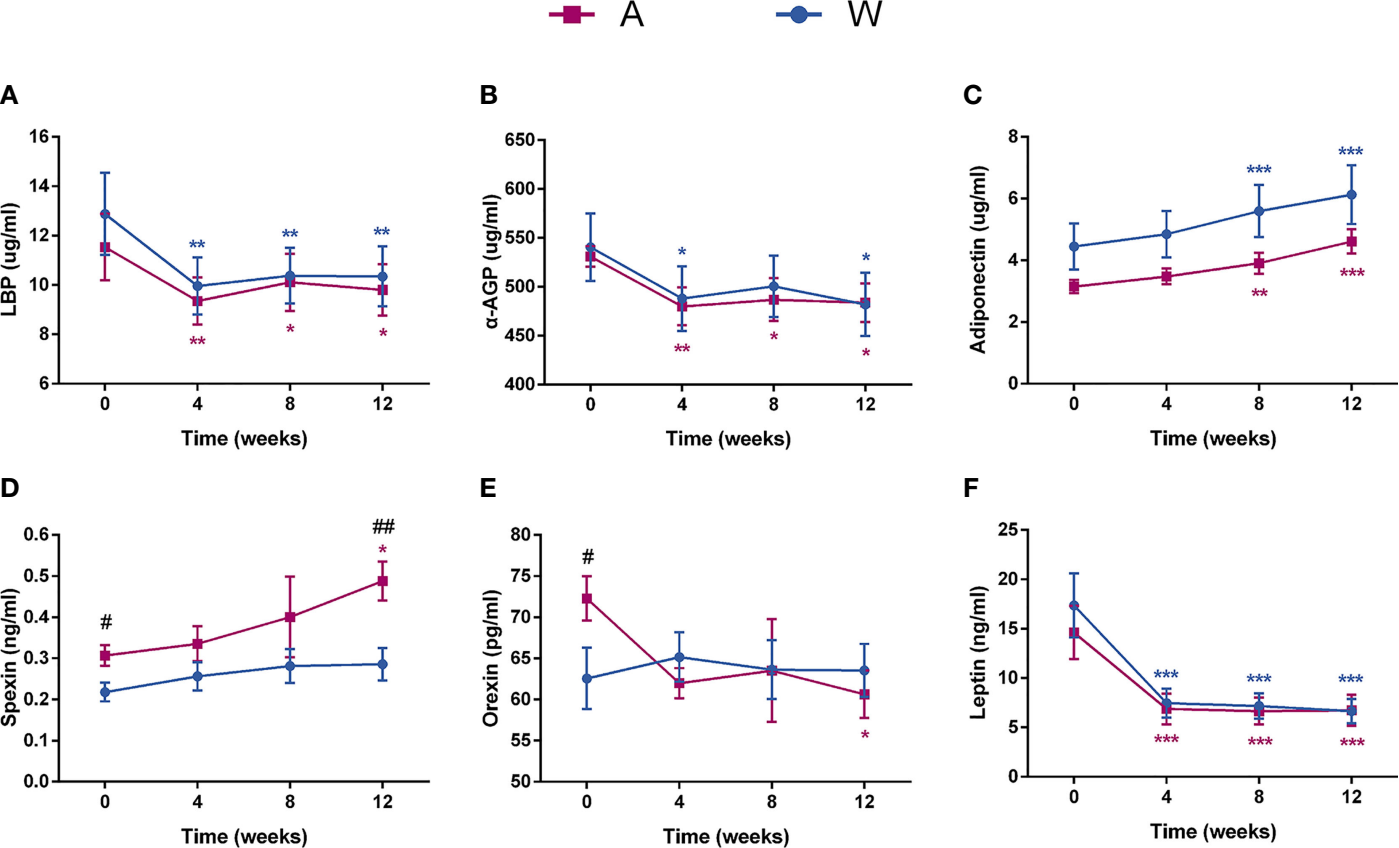
The state of chronic low-grade inflammation and the secretion of brain–gut peptides were altered in patients with PCOS after the intervention. Serum **(A)** LBP, **(B)** α-AGP, **(C)** adiponectin, **(D)** spexin, **(E)** orexin, and **(F)** leptin levels were tested by ELISA. Date expressed as mean ± SEM. *p < 0.05, **p < 0.01, and ***p < 0.001 for comparison with the week 0 value for the same group, red for A group and blue for W group. ^#^ *p* < 0.05 and ^##^ *p* < 0.01 for comparison of A group with W group value at the same time point. LBP, lipopolysaccharide -binding protein; α-AGP, α-1-acid glycoprotein.

Markers of inflammation including LBP and α-AGP decreased sharply at week 4 and maintained a stable level at the two afterward visits in the two groups (**Figures 3A, B** **)**. Meanwhile, the level of adiponectin, an anti-inflammatory cytokine, was significantly and steadily increased (**Figure 3C**). The level of spexin showed a significant trend of increase in the A group and was significantly higher than in the W group after intervention (**Figure 3D**). Orexin, a hypocretin peptide, was significantly decreased in the A group after intervention (**Figure 3E**). The serum leptin level of both groups decreased significantly at week 4 and maintain a stable level at the two afterward visits (**Figure 3F**).

### Altered Gut Microbiota by High-Fiber Diet or Combined With Acarbose

**Figure 4:**
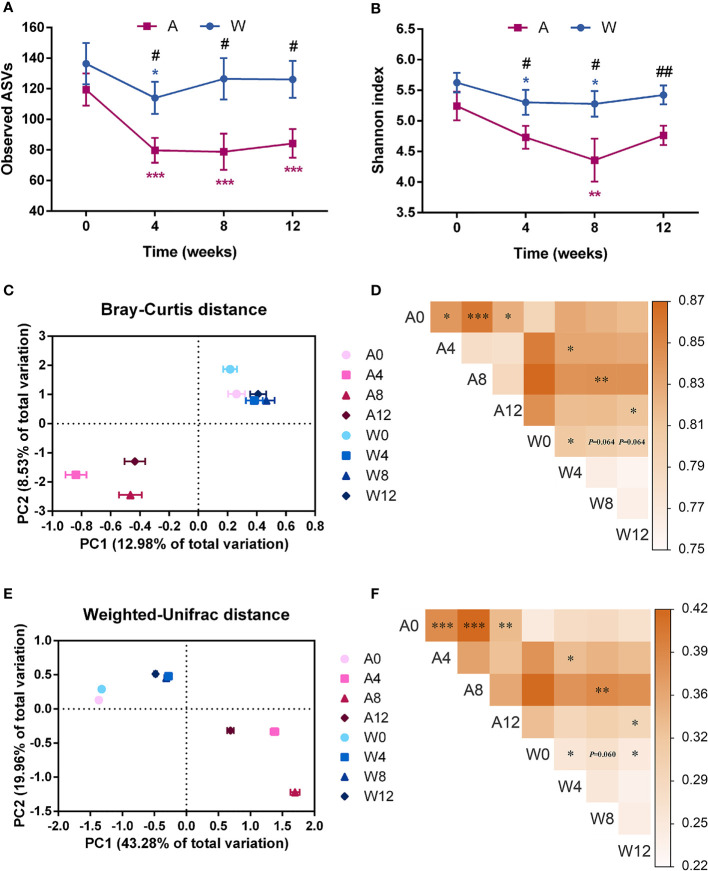
The gut microbial structure was altered in patients with PCOS after the intervention. Alpha diversity measured by **(A)** observed ASVs and **(B)** Shannon index. Date expressed as mean ± SEM. *p < 0.05, **p < 0.01, and ***p < 0.001 for comparison with the week 0 value for the same group, red for A group and blue for W group. ^#^ *p* < 0.05 and ^##^ *p* < 0.01 for comparison of A group with W group value at the same time point. Principal coordinates analysis (PCoA) performed on the basis of the **(C)** Bray–Curtis and **(E)** weighted-Unifrac distances showed the overall changes in gut microbial structure. PC1, principal coordinate 1; PC2, principal coordinate 2. PERMANOVA test was performed on the basis of the **(D)** Bray–Curtis and **(F)** weighted-Unifrac distances. The colors of the blocks indicate the distance, and the asterisks denote significant difference between two time points or different groups. *p < 0.05, **p < 0.01, and ***p < 0.001. A0, A4, A8, and A12: weeks 0, 4, 8, and 12 in the A group; W0, W4, W8, and W12: weeks 0, 4, 8, and 12 in the W group.

Gene sequencing on the V3–V4 regions of the 16S rRNA generated a dataset consisting of 2,178,945 high-quality reads and 1,731 ASVs, with an average of 21,789 ± 6,247 reads per sample. According to the observed ASVs and the Shannon index, the richness of gut microbiota was significantly reduced in both groups and lower in the A group than in the W group after intervention (**Figures 4A, B** **)**.

In the context of beta diversity based on Bray–Curtis (**Figures 4C, D** **)** and weighted-Unifrac (**Figures 4E, F** **)** distances, the overall microbial structure showed no difference between the two groups at baseline. Significant alterations were observed from baseline to week 4 in both groups, with no further changes afterwards. These results showed that the two sets of intervention both modulated the gut microbiota of patients with PCOS, and such modulation was stable. At weeks 4 until 12, especially at week 8, significant separation between the two groups reflected a distinct modulatory effect of the high-fiber diet combined with acarbose on the gut microbiota, compared to the simple high-fiber diet.

**Figure 5:**
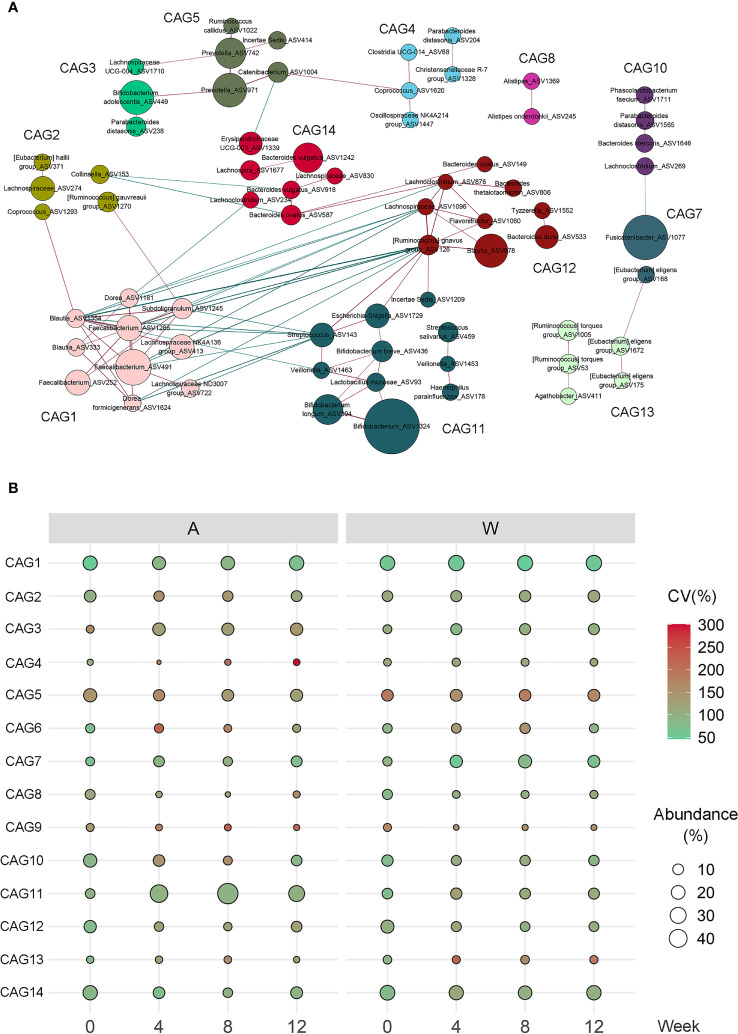
Alterations in the abundance of CAGs in response to the intervention. **(A)** Co-abundance groups interaction network displays the interaction between different CAGs. Node size represents the average abundance of each ASV. Lines between the nodes indicate correlation (green = negative, red = positive), with width of the lines representing the correlation magnitude. Correlations with absolute values <0.4 are not shown here. **(B)** Bubble plot shows the variation in the average abundance of CAGs during the intervention. The size and color of the circles represent the average abundance and coefficient of variance (CV) of each CAG, respectively.

As bacteria work together as a coherent functional group in the gut ecosystem (31), we constructed a co-abundance network between the 132 ASVs, which were shared by at least 20% of all samples, and clustered the ASVs into 14 CAGs (**Figure 5A** and **Supplementary Table S3**). Of these, three CAGs, including CAG3, CAG7, and CAG11, were significantly increased in the A group, while two CAGs, including CAG3 and CAG7, were significantly increased in the W group. Six CAGs, including CAG4, CAG8, CAG9, CAG10, CAG12, and CAG14, were significantly decreased in the A group, while four CAGs, including CAG8, CAG9, CAG10, and CAG12, were significantly decreased in the W group. Six CAGs, including CAG4, CAG6, CAG7, CAG8, CAG11, and CAG14, responded to the differentiation between the two groups at weeks 4, 8, and 12, five of which were also significantly altered in the A or W group. Max member of CAGs differed at week 4 between the two groups. CAG11 remain different at weeks 4, 8, and 12 (**Figure 5B**).

Among these CAGs, CAG3, and CAG11, which were enriched in the A group, were the dominant CAGs with ASVs from *Bifidobacterium* and *Lactobacillus*. CAG7 was 66.3% comprised predominantly of ASV1077 from *Fusicatenibacter*. CAG8, inhibited in both groups, was comprised predominantly of *Bacteroides vulgatus* and *Alistipes*. The other CAGs, inhibited in both groups, included CAG12 composed of ASVs from *Bacteroides* and *Blautia*, CAG10 composed of ASVs from *Lachnospira* and *Roseburia*, and CAG9 composed of ASVs from *Bilophila*. CAG14, inhibited only in the A group, contained three ASVs from *B. vulgatus*.

### Associations Between Gut Microbiota and Clinical Parameters in Patients With PCOS

**Figure 6:**
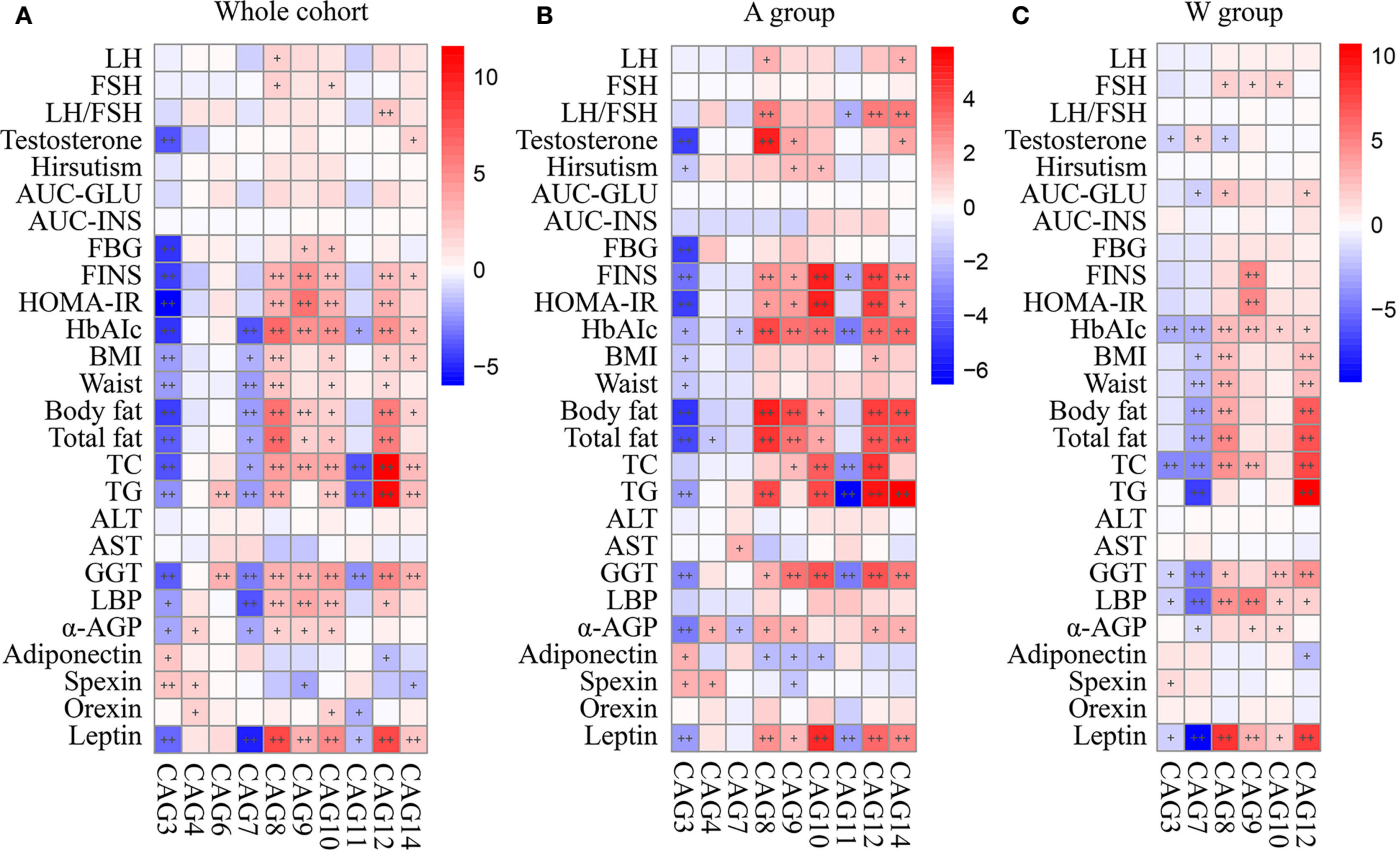
Correlations between primary clinical parameters and altered CAGs in **(A)** the whole cohort, **(B)** A group, and **(C)** W group. In the heat map, spots color represents R-value of MaAslin2 correlation between each CAG and clinical parameter. ^+^FDR < 0.25 and ^++^FDR < 0.1. LH, luteinizing hormone; FSH, follicular stimulating hormone; AUC-Glucose, the glucose area under the curve of OGTT; AUC-Insulin, the insulin area under the curve of OGTT; FBG, fasting blood glucose; FINS, fasting plasma insulin; HOMA-IR, homeostasis model assessment for insulin resistance index; HbA1c, hemoglobin A1c; BMI, body mass index; TC, total cholesterol; TG, triglyceride; ALT, alanine aminotransferase; AST, aspartate transaminase; GGT, γ-glutamyltransferase; LBP, lipopolysaccharide-binding protein; α-AGP, α-1-acid glycoprotein.

To explore the relationships between the altered CAGs and host clinical phenotypes, we used MaAslin2 to get the correlations between the CAGs and 26 clinical parameters in different cohorts (**Figure 6**). When examining the whole cohort or A group separately, we saw that the CAGs enriched by the intervention were negatively correlated with the disease phenotypes, such as the LH/FSH ratio, testosterone, metabolic parameters including FINS and homeostasis model assessment-insulin resistance (HOMA-IR), α-AGP, leptin, and orexin, and positively correlated with adiponectin and spexin, while the CAGs inhibited had an opposite trend (**Figures 6A, B** **)**. Compared with the A group, the CAGs altered in the W group had the same trend but with weaker correlation with the disease phenotypes, mainly including the LH/FSH ratio, testosterone, the hirsutism score, FINS, and HOMA-IR (**Figure 6C**).

## Discussion

PCOS is a clinical syndrome with metabolic, endocrine, and reproductive dysfunctions. Dietary interventions as a first-line treatment for patients with PCOS have been evaluated, but the optimal diet pattern is uncertain. Effective treatments for PCOS are still lacking. Our clinical trial showed that high-fiber diet or combined with acarbose alleviate PCOS by regulating gut microbiota. In this trial, we conducted a 12-week clinical intervention in patients with PCOS, achieved the improvement of reproductive endocrine and glycolipid metabolic disorders, and promoted fertility *via* intervention of no matter simple high-fiber diet or combined with acarbose. As far as we know, this trial is the first gut microbiota-targeted nutritional intervention combined with oral hypoglycemic agent for the patients with PCOS. The dysbiosis of gut microbiota was remodeled by a high-fiber diet, probiotics were enriched, and noxious bacteria were inhibited, thus leading to the changes in pH level and short-chain fatty acids (SCFAs) production (**Supplementary Figure S1**). It is worth noting that a high-fiber diet combined with acarbose showed the better improvement of reproductive endocrine disorders, hyperinsulinemia and insulin resistance, and more optimal composition of the gut microbiota. Furthermore, the changes in gut microbiota and SCFAs were associated with sex hormonal and metabolic parameters, inflammatory factors, and brain–gut peptides, especially in the A group.

Obesity and insulin resistance are strongly implicated in the etiology of PCOS, and lifestyle modification, especially dietary modification and exercise, is the primary method to improve insulin sensitivity (3). Weight loss leads to the improvements in the health of overweight/obese women with PCOS (32, 33). Dietary fiber supplementation has a definite effect on weight loss (34). The intake of dietary fibers is also associated with insulin resistance, hyperandrogenism, and body composition in patients with PCOS (7, 35). In our study, both of the two groups were given a high-fiber diet and showed a significant reduction in BMI, which may alleviate the pathophysiological defects of PCOS. Moreover, the structure of gut microbiota changed significantly after high-fiber diet intervention compared with baseline. The dietary fibers we provided was a single staple food, and this single diet led to the reduction in richness of gut microbiota. Our data suggest that optimal structure of the gut microbiota rather than simple greater overall diversity implies better health, which is consistent with our previous study (18). Besides, dietary fibers could promote the production of key metabolites such as SCFAs, impacting host–microbe interactions (18, 36), and this mechanism might explain our results about the SCFAs. Furthermore, SCFAs could combat obesity and insulin resistance, a core pathogenic factor of PCOS, by brain–gut peptides (37). Therefore, it suggests that dietary fibers may alleviate the clinical phenotypes of PCOS, and the gut microbiota may play an important role in the process.

Acarbose could push more carbohydrates into the large intestine for fermentation. This mechanism confirmed the modulation of acarbose on gut microbiota (38). However, a previous study found that acarbose combined with another kind of high-fiber diet promoted more probiotics than acarbose alone (18), in relative agreement with our finding that acarbose could strengthen the modulation of dietary fibers on gut microbiota. We found that the high-fiber diet combined with acarbose could better improve the hyperandrogenism and insulin resistance of host, which suggested that acarbose alleviates PCOS by the two core pathogenic factors. However, it seemed to be due to the participants neglect of their diet from the third month; these improvements were rebounded slightly at the end of our intervention, and this trend was consistent with changes in gut microbiota. However, the specific mechanism remains to be further studied.

Multiple inflammatory factors and brain–gut peptides may be involved in the development of PCOS. LBP, known as LPS-binding protein, could affect insulin sensitivity and lead to IR (39). Zhu et al. found that LBP level in patients with PCOS was significantly increased, irrespective of body mass (40). α-AGP and adiponectin are adipokines, belonging to the family of cytokines and inflammatory proteins that are produced by adipose tissue (41, 42). α-AGP may modulate energy homeostasis and food intake by interacting with leptin receptor (43). In PCOS, α-AGP level was higher and correlated with biomarkers of adiposity and total testosterone (44). Adiponectin mediates insulin-sensitizing effect through binding to its receptors AdipoR1 and AdipoR2 (42). Spexin is a novel hypothalamic neuropeptide that exerts inhibitory effect on feeding and maintains reproductive function *via* gonadotropin-releasing hormone neurons (45–47). The decreased level of spexin is associated with sex hormonal and metabolic disorders of PCOS (48). Orexin is another hypothalamic neuropeptide and has been demonstrated to evoke hyperphagia and obesity (49). Leptin is involved in gut–brain axis, thus regulating appetite and energy metabolism (50, 51). Leptin, adiponectin, and the leptin/adiponectin ratio are correlated with insulin resistance (52). Our study showed that patients of PCOS had a significant decrease in LBP, α-AGP, and leptin, and significant increase in adiponectin after the intervention. Spexin significantly increased and orexin significantly decreased in the intervention added of acarbose. Our results indicate that the intervention may affect the levels of inflammatory factors and brain–gut peptides and thus alleviate the clinical phenotypes of PCOS.

A previous study slightly focused on the beneficial effects of prebiotics in patients with PCOS. Dietary fibers and acarbose may affect the gut microbiota structure in the form of CAGs, rather than single bacteria. Here, we observed that CAGs, comprised predominantly of *Bifidobacterium* and *Fusicatenibacter*, were enriched after the intervention in both of the two groups. Ren et al. (53) reported that the decrease in the abundance of *Fusicatenibacter* in non-alcoholic fatty liver disease was linked with the metabolism disorders such as insulin resistance. According to the report by Takada et al. (54), *Fusicatenibacter* could ferment carbohydrates to produce SCFAs. It should be noted that high-fiber diet combined with acarbose enriched large numbers of *Bifidobacterium* and a strain of *Lactobacillus* than simple high-fiber diet. *Bifidobacterium* could ferment carbohydrates including mannose, fructose, sucrose, and lactose (55). More studies showed that the fermentation of prebiotics including inulin by *Bifidobacterium* and *Lactobacillus* promoted host health (56, 57). The results of our study suggest that acarbose promote more carbohydrates into the large intestine for *Bifidobacterium* and *Lactobacillus* consumption and thus lead to the enrichment of ASVs in the A group. According to previous studies, *Bifidobacterium* and *Lactobacillus* genera are rich in acetic-acid-producing bacteria, which is in agreement with the acetic change in our result (**Supplemetary Figure S1**). In our study, CAG3 and CAG11, mainly containing *Bifidobacterium*, had a negative correlation with the levels of LH/FSH ratio, testosterone, glycolipid metabolism, inflammatory factors, orexin, and leptin, while they were positive correlated with the levels of adiponectin and spexin. Zhang et al. (14) proved a direct evidence that *Bifidobacterium lactis V9* regulates the levels of sex hormones in PCOS *via* the gut–brain axis. CAG7, mainly containing *Fusicatenibacter*, had a negative correlation with the glycolipid metabolism parameters, inflammatory factors, and leptin. These results suggest that the enrichment of these bacteria may alter the chronic metabolic inflammation and brain–gut peptides of host and thus alleviate PCOS by affecting the SCFAs production.

On the other hand, our intervention also decreased the relative abundance of several CAGs, comprised predominantly of noxious bacteria, such as *Bacteroides*, mainly of *B. vulgatus*, and *Alistipes*, *Bilophila*, *Blautia*, *Lachnospira*, and *Roseburia*. It should be noted that a high-fiber diet combined with acarbose inhibited more ASVs from *B. vulgatus* than a simple high-fiber diet. CAG8 and CAG14, mainly containing *B. vulgatus*, were stronger inhibited in the A group and had a positive correlation with the levels of LH/FSH ratio, testosterone, hyperinsulinemia, insulin resistance, BMI, inflammatory factors, and leptin, while they were negatively correlated with the levels of adiponectin and spexin. Hence, it can be seen that acarbose strengthened the modulation of gut microbiota by a high-fiber diet and might better alleviate PCOS through the two core pathogenic factors, namely, hyperandrogenism and insulin resistance. *Bacteroides* in the human intestine are Gram-negative bacteria, a kind of famous LPS-producing bacteria (58). The positive correlation of *Bacteroides* and metabolism disorders in PCOS from a cross-sectional study confirmed our result (10). Sun et al. (59) and Jiang et al. (60) elaborated our results in the mechanism; they pointed out that *Bacteroides fragilis* and *B. vulgatus* affected the host metabolic disorders and inflammation of T2DM and PCOS *via* the gut microbiota–bile acid axis. Besides, CAG8 also contained ASVs from *Alistipes*. *Alistipes*, another kind of common LPS-producing bacteria, is associated with obesity and inflammation and can aggravate metabolism disorders induced by high-fat diet (61). Other CAGs, such as CAG9, CAG10, and CAG12, containing ASVs from *Bacteroides*, *Bilophila*, *Blautia*, and *Roseburia*, were inhibited in both of the two groups and had a positive correlation with a series of glycolipid metabolism parameters, inflammatory factors, orexin, and leptin, while they were negatively correlated with adiponectin and spexin. *Bilophilia*, another common LPS-producing bacteria, could promote higher inflammation and bile acid dysmetabolism, thus aggravating the metabolic dysfunctions induced by high-fat diet (62). Lin et al. reported an increase in the abundance of *Blautia* genera, and *Roseburia* genera are correlated with the alterations of bile acids in obesity (63). These results suggest that the inhibition of these bacteria may improve the chronic metabolic inflammation by affecting the LPS production and bile acid metabolism, thus alleviating PCOS.

Our study was a pilot exploration of dietary or drug interventions targeting gut microbiota in patients with PCOS. The downside of our study is the small sample size. Due to the strict quality control of the experimental process, the dietary intervention programs of all subjects were consistent, and the changes in physiological and biochemical parameters and gut microbiota showed the same trend. Further multicenter, placebo-controlled, double-blind studies, metabonomic studies, and fecal microbiota transplantation in germ-free mice are needed to be performed to explore more mechanisms of how the gut microbiota modulation alleviate PCOS.

In summary, our work suggested that high-fiber diet could alleviate the chronic metabolic inflammation, reproductive function, and brain–gut peptides secretion of patients with PCOS, and a high-fiber diet combined with acarbose could better improve the PCOS clinical phenotypes. Meanwhile, the remodeling of gut microbiota by our intervention may play an important role in these improvements. Gut microbiota targeted intervention may present a novel approach for the treatment of PCOS in clinical practice.

## Data Availability Statement

The original contributions presented in the study are included in the article/**Supplementary Material**, further inquiries can be directed to the corresponding authors.

## Ethics Statement

The studies involving human participants were reviewed and approved by the Human Research Ethics Committee of Shanghai General Hospital. Written informed consent to participate in this study was provided by the participants’ legal guardian/next of kin.

## Author Contributions

XWa performed clinical research, DNA extraction and sequencing, and the bioinformatics and statistical analysis. TX performed the bioinformatics and statistical analysis. RL performed DNA extraction and SCFA measurements. GW performed the bioinformatics analysis. LG and YLi collected clinical data. YZ, XWe, and YLu performed statistical analysis. FZ and JS performed clinical research. HF performed nutritional intervention. LZ, YP, and CZ designed the study and performed quality control. XD designed the study, performed clinical research, and wrote the manuscript. All authors contributed to the article and approved the submitted version.

## Funding

The study was supported by the National Natural Science Foundation of China (81870594); Shanghai Jiao Tong University Research Funding on Medical, Engineering Interdisciplinary Project (YG2019GD05); Clinical Research Plan of SHDC (SHDC2020CR1016B); Multi-center Clinical Research Project of Shanghai Jiao Tong University School of Medicine (DLY201824); The Third Round Cooperation Project of Songjiang District Municipal Health Commission (0702N18003).

## Conflict of Interest

The authors declare that the research was conducted in the absence of any commercial or financial relationships that could be construed as a potential conflict of interest.

## Publisher’s Note

All claims expressed in this article are solely those of the authors and do not necessarily represent those of their affiliated organizations, or those of the publisher, the editors and the reviewers. Any product that may be evaluated in this article, or claim that may be made by its manufacturer, is not guaranteed or endorsed by the publisher.
