# Supplementary material for: High-Fiber Diet or Combined With Acarbose Alleviates Heterogeneous Phenotypes of Polycystic Ovary Syndrome by Regulating Gut Microbiota
Source: Front Endocrinol (Lausanne). 2022 Feb 2;12:806331. doi: 10.3389/fendo.2021.806331 (PMC8847200; doi:10.3389/fendo.2021.806331)
Supplement: Supplementary file 1 [file DataSheet_1.docx]

***Supplementary Material***

This file includes:

Materials and Methods

Supplementary Text

Figs. S1 to S2

Tables S1 to S2

References

**Materials and Methods**

**Fecal short-chain fatty acid (SCFA) measurement**

Fecal SCFA concentrations were measured by gas chromatography/mass spectrometry (GC/MS), as described previously ([1](#_ENREF_1)). Briefly, 500 mg fecal samples were homogenized with 1.0 mL of phosphate buffer (pH 7.3) and centrifuged at 4°C at 16,000 x g for 15 min. The pH value of the supernatants was measured, and then the supernatants were filtered through a 0.22 µm nylon filter (EMD Millipore). 200 µL supernatants was acidified with 100 ul 50% (v/v) sulfuric acid. After vortexed and stood for 2 min, 0.4 mL diethyl ether was added to extract the organic acids, and supernatants were measured by GC on an Agilent 7890A (Agilent Technologies, CA, USA) equipped with a flame ionization detector, capillary columns (Hewlett-Packard, CA, United States) and GC ChemStation software. In this paper, the term “Total SCFAs” means the sum of acetic acid, propionic acid, butyric acid, isobutyric acid, valeric acid and isovaleric acid concentrations.

**Supplementary Text**

It should be emphasized that the women with PCOS recruited were infertile. Although we informed contraception was needed in our intervention, some of the participants still had unplanned pregnancy during the intervention. And a total of 4 women in A group and 4 women in W group successfully delivered within the next two years of follow-up, especially one of them in the A group had multiple failures of assisted reproductive technology in the past.

The A group had a significant reduction in the fecal pH value at Week 8 compared with baseline (Figure S1A). The relative concentration of acetic acid increased significantly in both groups, and was higher in the A group (Figure S1C). The relative concentrations of isobutyric acid and isovaleric acid, markers of protein fermentation, were significantly decreased in both groups and significantly lower in the A group than the W group (Figure S1F and S1H). The relative concentrations of propionic acid and valeric acid were significantly decreased only in the A group (Figure S1D and S1G). Thus, the gut microbiota tended to ferment carbohydrates more than proteins after the intervention.

The relative concentration of acetic acid showed significantly negative correlations with LH, metabolic parameters, inflammatory factors, orexin and leptin. Butyric acid was negatively correlated with HbA1c and positively correlated with spexin. Propanoic acid, isobutyric acid, valeric acid and isovaleric acid showed the opposite trend with clinical parameters, including HOMA-IR (Figure S2A). When examining the A group and W group separately, we saw the correlations between SCFAs and reproductive endocrine parameters like testosterone and LH/FSH, metabolic parameters and mediators of adiponectin and leptin (Figure S2B). In the W group, only the correlation of SCFAs and metabolic parameters remained significant (Figure S2C).

**Supplementary Figures**


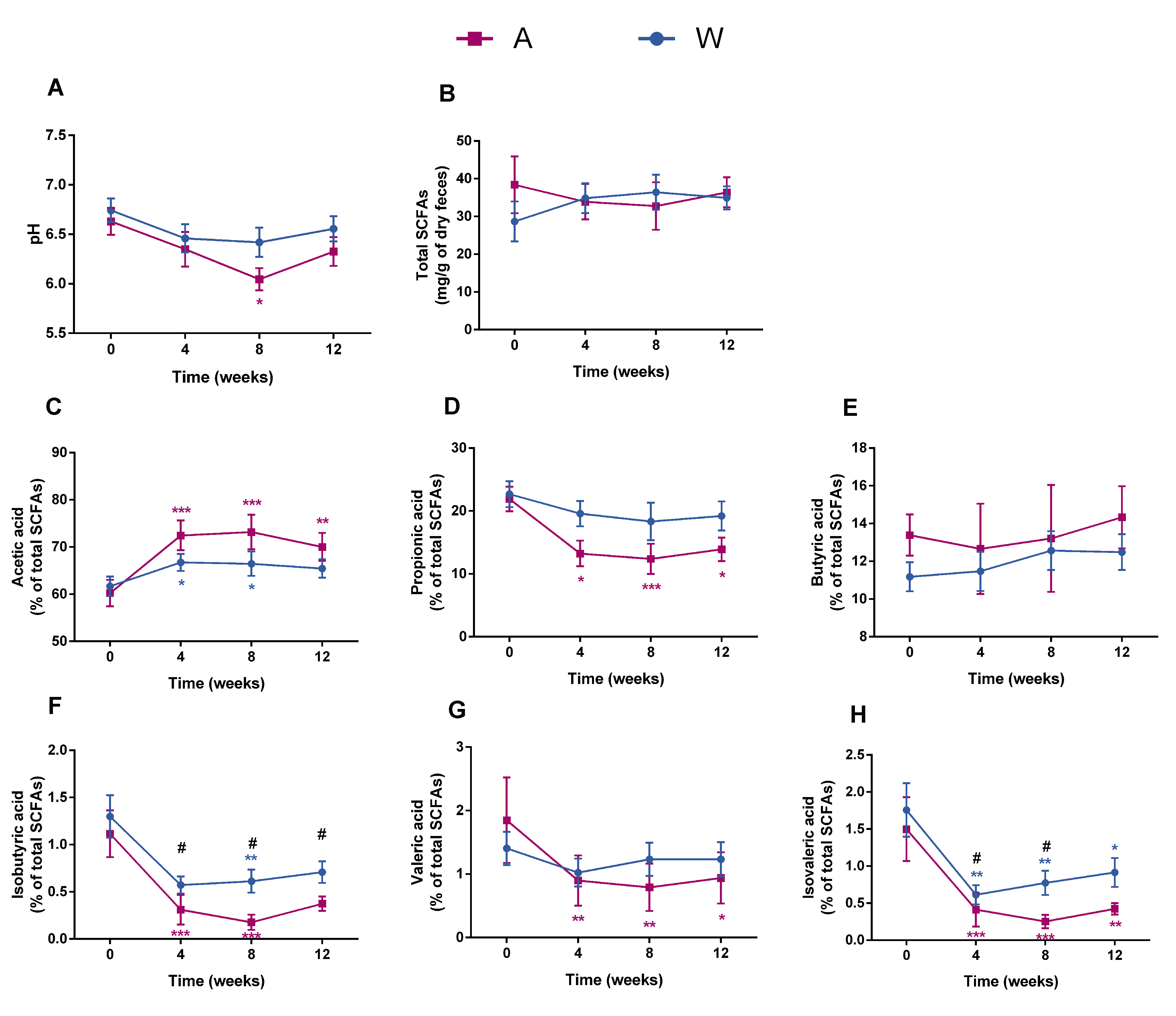


Figure S1. The relative concentrations of short chain fatty acids (SCFAs) were changed in patients with PCOS after the intervention. Relative concentrations of A) fecal water pH value, B) total SCFAs, C) acetic acid, D) propionic acid, E) butyric acid, F) isobutyric acid, G) valeric acid and H) isovaleric acid are shown. Date expressed as mean ± SEM. **P* < 0.05, ***P* <0.01, and ****P* < 0.001 for comparison with the Week 0 value for the same group, red for A group and blue for W group. #*P* < 0.05 for comparison of A group with W group value at the same time point.


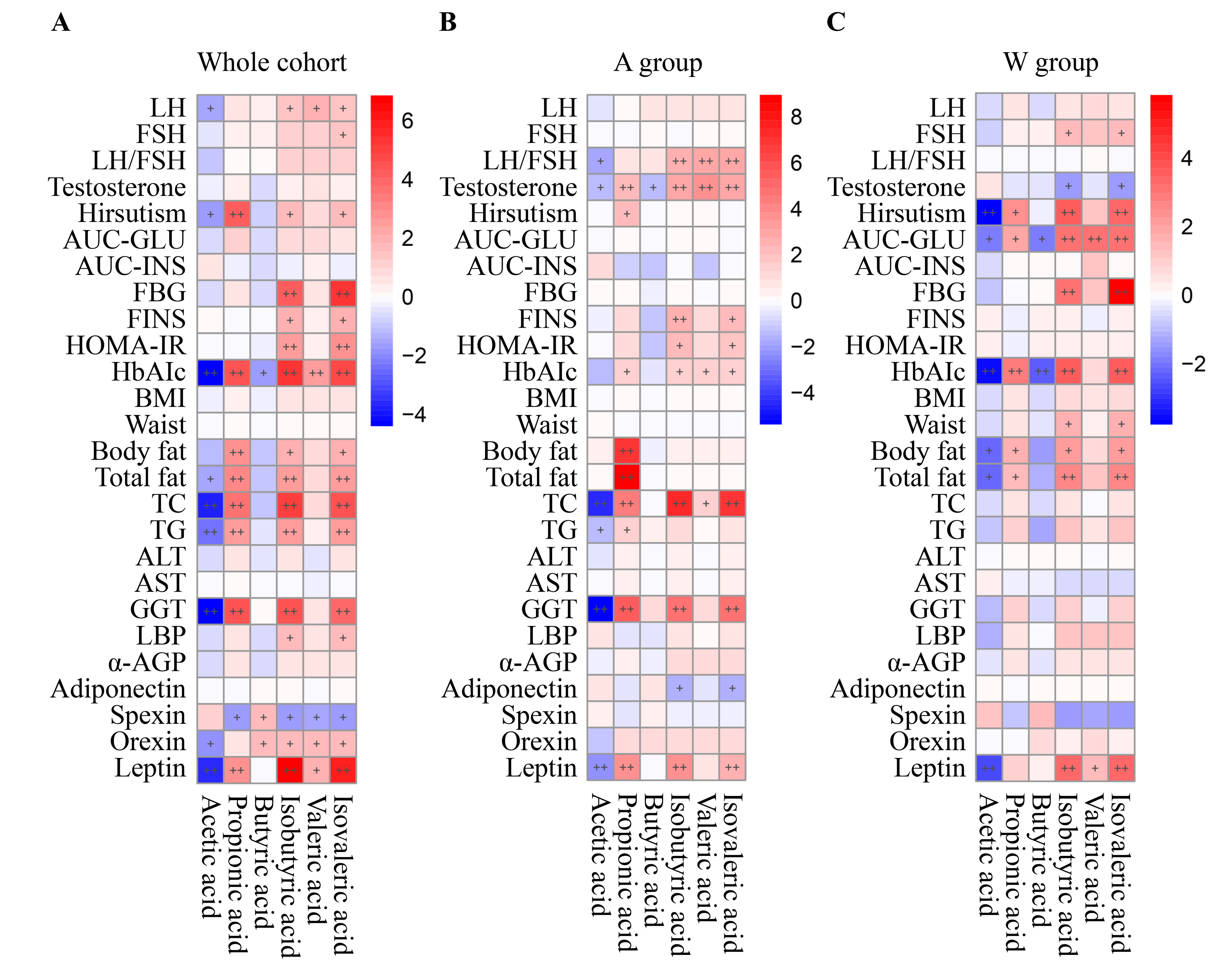


Figure S2. Correlations between the primary clinical parameters and relative concentrations of SCFA in A) the whole cohort, B) A group and C) W group. In the heat map, spots color represents R value of MaAslin2 correlations between SCFA and clinical parameter. +FDR<0.25 and ++FDR<0.1. Abbreviations: LH, luteinizing hormone; FSH, follicular stimulating hormone; AUC-Glucose, the glucose area under the curve of OGTT; AUC-Insulin, the insulin area under the curve of OGTT; FBG, fasting blood glucose; FINS, fasting plasma insulin; HOMA-IR, homeostasis model assessment for insulin resistance index; HbA1c, hemoglobin A1c; BMI, body mass index; TC, total cholesterol; TG, triglyceride; ALT, alanine aminotransferase; AST, aspartate transaminase; GGT, γ-glutamyltransferase; LBP, lipopolysaccharide-binding protein; α-AGP, α-1-acid glycoprotein.

**Supplementary Tables**

| **TABLE S1 \|** Change of clinical parameters, inflammatory factors and brain gut peptides after the intervention in the two groups. | | | | | | | | | |
| --- | --- | --- | --- | --- | --- | --- | --- | --- | --- |
| **Characteristic** | **Group** | **Week** | | | | **P value (A vs W)** | | | |
|  |  | **0** | **4** | **8** | **12** | **0** | **4** | **8** | **12** |
| Age, years | A (11) | 27.1±5.9 | / | / | / | / | / | / | / |
|  | W (14) | 28.0±6.3 | / | / | / |  |  |  |  |
| **Reproductive endocrine parameters** | | | | |  |  |  |  |  |
| LH, IU/L | A | 15.1 (8.5, 19.4) | 11.3 (6.6, 15.6) | 6.4 (2.6, 10.9) ** | 8.9 (4.1, 9.7) * | 0.679 | 0.476 | 0.044 | 0.491 |
|  | W | 10.9 (6.9, 19.8) | 8.9 (4.6, 13.0) | 9.2 (6.4, 16.1) | 7.2 (5.0, 12.6) |  |  |  |  |
| FSH, IU/L | A | 7.3 (4.4, 7.8) | 6.0 (4.0, 7.3) | 3.8 (1.7, 6.4) * | 6.5 (3.9, 7.2) | 0.395 | 0.641 | 0.286 | 0.087 |
|  | W | 7.3 (4.6, 8.9) | 4.9 (4.0, 7.0) | 4.3 (3.5, 7.0) | 7.6 (5.2, 8.8) |  |  |  |  |
| LH/FSH | A | 2.2±0.8 | 1.9±0.8 | 1.5±0.4 | 1.3±0.6 * | 0.566 | 0.289 | 0.050 | 0.883 |
|  | W | 2.1±1.2 | 1.6±0.7 | 2.4±1.2 | 1.3±0.6 |  |  |  |  |
| Testosterone, ng/dL | A | 176.2 (129.2, 215.88) | 138.4 (72.0, 180.4) ** | 105.6 (83.2, 126.1) *** | 141.2 (121.2, 189.5) | 0.265 | 0.800 | 0.712 | 0.426 |
|  | W | 132.6 (60.9, 211.0) | 112.6 (65.5, 174.9) | 126.0 (78.2, 158.1) | 176.4 (96.6, 260.5) * |  |  |  |  |
| Hirsutism score | A | 10.0 (7.0, 20.0) | / | / | 6.0 (5.5, 8.5) ** | 0.902 | / | / | 0.440 |
|  | W | 13.5 (6.5, 14.75) | / | / | 7.0 (6.0, 11.25) |  |  |  |  |
| Ovarian volume, mL | A | 9.9 (7.4, 14.5) | / | / | 9.6 (7.8, 12.2) | 0.488 | / | / | 0.897 |
|  | W | 9.6 (7.5, 12.9) | / | / | 11.4 (6.5, 12.0) |  |  |  |  |
| Immature follicles number | A | 15.9±3.1 | / | / | 13.5±3.8 | 0.933 | / | / | 0.660 |
|  | W | 15.1±4.7 | / | / | 14.8±5.9 |  |  |  |  |
| **Metabolic parameters** | | | | |  |  |  |  |  |
| FBG, mmol/L | A | 4.3±0.6 | 3.8±0.4 | 4.1±0.3 | 4.1±0.3 | 0.082 | 0.947 | 0.005 | 0.009 |
|  | W | 4.6±0.5 | 3.9±0.5 *** | 4.5±0.4 | 4.5±0.4 |  |  |  |  |
| 2h BG, mmol/L | A | 7.1±1.8 | / | / | 5.9±1.6 | 0.599 | / | / | 0.182 |
|  | W | 7.0±1.6 | / | / | 5.3±2.1 *** |  |  |  |  |
| AUC-Glucose, mmol/L/min | A | 911.9±172.5 | / | / | 808.1±183.5 | 0.724 | / | / | 0.300 |
|  | W | 880.8±151.8 | / | / | 747.3±209.0 ** |  |  |  |  |
| FINS, pmol/L | A | 69.5 (36.2, 105.0) | 30.0 (25.0, 63.5) ** | 30.0 (18.5, 35.8) *** | 38.5 (20.5, 85.5) * | 0.598 | 0.512 | 0.074 | 0.437 |
|  | W | 48.0 (38.5, 85.5) | 43.0 (30.5, 68.5) | 41.0 (28.0, 69.0) | 25.0 (17.5, 55.5) ** |  |  |  |  |
| 2h INS, pmol/L | A | 594.0 (316.5, 1629.0) | / | / | 346.0 (124.5, 984.5) | 0.940 | / | / | 0.760 |
|  | W | 616.5 (389.5, 1179.5) | / | / | 411.0 (236.5, 502.8) ** |  |  |  |  |
| AUC-Insulin, pmol/L/min | A | 7346.0 (5571.0, 14984.0) | / | / | 5022.5 (3530.8, 11993.3) | 0.528 | / | / | 0.528 |
|  | W | 10454.0 (6234.0, 13612.0) | / | / | 7378.0 (5314.0, 9129.5) |  |  |  |  |
| HOMA-IR | A | 2.0 (0.9, 2.7) | 0.7 (0.6, 1.7) ** | 0.8 (0.5, 0.9) *** | 0.9 (0.5, 2.2) * | 0.821 | 0.436 | 0.057 | 0.557 |
|  | W | 1.5 (1.0, 2.7) | 1.1 (0.7, 1.7) * | 1.2 (0.8, 2.2) | 0.7 (0.5, 1.7) ** |  |  |  |  |
| HbA1c, % | A | 5.7±0.3 | / | / | 5.1±0.5 ** | 0.490 | / | / | 0.258 |
|  | W | 5.7±0.6 | / | / | 5.1±0.2 ** |  |  |  |  |
| BMI, kg/m^2^ | A | 28.1±5.1 | 26.3±4.7 *** | 25.7±4.5 *** | 25±4.1 *** | 0.315 | 0.530 | 0.399 | 0.462 |
|  | W | 26±6.1 | 24.9±5.7 ** | 24±5.2 *** | 23.7±5.1 *** |  |  |  |  |
| Waist, cm | A | 90.2±13.2 | 87±11.3 | 84.6±13 ** | 84±12.1 ** | 0.426 | 0.380 | 0.249 | 0.169 |
|  | W | 86±15.4 | 82.8±15.4 | 78.2±11.4 *** | 76.9±11.1 *** |  |  |  |  |
| Body fat, % | A | 44.4±4.6 | / | / | 38.1±6.7 *** | 0.459 | / | / | 0.395 |
|  | W | 41.7±8.4 | / | / | 35±8.8 *** |  |  |  |  |
| Total fat, % | A | 41.3±4.3 | / | / | 36±5.5 *** | 0.599 | / | / | 0.510 |
|  | W | 39.5±6.8 | / | / | 34.2±7.1 *** |  |  |  |  |
| TC, mmol/L | A | 4.4±0.8 | 3.6±0.5 *** | 3.7±0.7 *** | 3.8±0.6 *** | 0.596 | 0.117 | 0.406 | 0.682 |
|  | W | 4.6±0.8 | 4.0±0.5 ** | 4.1±0.7 * | 4.0±0.6 ** |  |  |  |  |
| TG, mmol/L | A | 1.0 (0.7, 1.2) | 0.8 (0.6, 1.1) | 0.6 (0.6, 0.9) *** | 0.6 (0.5, 0.9) ** | 0.863 | 0.964 | 0.706 | 0.940 |
|  | W | 1.1 (0.6, 1.4) | 0.8 (0.5, 1.1) * | 0.8 (0.6, 1.1) * | 0.6 (0.5, 0.9) ** |  |  |  |  |
| ALT, U/L | A | 17.5 (11.8, 27.2) | 17.3 (11.1, 24.7) | 16.9 (11.5, 24.1) | 13.5 (12.3, 18.5) | 0.925 | 0.525 | 0.395 | 0.366 |
|  | W | 17.5 (13.5, 22.0) | 18.9 (16.6, 25.2) | 18.3 (13.9, 23.3) | 16.7 (13.2, 20.4) |  |  |  |  |
| AST, U/L | A | 16.6 (15.4, 21.6) | 19.7 (17.0, 24.0) | 20.2 (17.7, 21.8) | 19.5 (14.3, 23.3) | 0.787 | 0.598 | 0.832 | 0.898 |
|  | W | 18.6 (15.2, 19.8) | 21.3 (18.7, 23.3) | 20.2 (17.6, 22.9) | 19.9 (16.9, 21.3) |  |  |  |  |
| GGT, U/L | A | 16.6 (13.4, 19.5) | 13.0 (9.2, 15.6) *** | 11.4 (8.7, 15.0) *** | 10.9 (8.9, 13.1) *** | 0.291 | 0.427 | 0.555 | 0.319 |
|  | W | 14.4 (10.8, 17.5) | 12.0 (7.8, 13.4) ** | 11.3 (8.6, 12.6) ** | 10.2 (7.3, 11.0) *** |  |  |  |  |
| **Inflammatory indicators and brain-gut peptides** | | | | |  |  |  |  |  |
| LBP, ug/mL | A | 11.5±4.5 | 9.4±3.2 ** | 10.1±3.8 * | 9.8±3.5 * | 0.673 | 0.793 | 0.597 | 0.917 |
|  | W | 12.9±6.2 | 10±4.3 ** | 10.4±4.2 ** | 10.3±4.5 ** |  |  |  |  |
| α-AGP, ug/mL | A | 531±35.1 | 480±64.3 ** | 486.8±73 * | 483.8±65.2 * | 0.759 | 0.834 | 0.793 | 0.967 |
|  | W | 540.5±128.9 | 488±123.4 * | 500.4±117.7 | 482±120.6 * |  |  |  |  |
| Adiponectin, ug/mL | A | 3.1 (2.7, 3.7) | 3.3 (3.0, 4.0) | 4.0 (3.0, 4.5) * | 4.3 (3.6, 5.9) *** | 0.491 | 0.395 | 0.262 | 0.338 |
|  | W | 4.0 (2.0, 6.5) | 4.5 (2.6, 6.7) | 5.3 (3.4, 6.4) *** | 5.3 (3.5, 8.0) *** |  |  |  |  |
| Spexin, ng/mL | A | 0.3 (0.2, 0.4) | 0.3 (0.3, 0.4) | 0.3 (0.2, 0.4) | 0.5 (0.3, 0.6) * | 0.011 | 0.182 | 0.182 | 0.003 |
|  | W | 0.2 (0.2, 0.3) | 0.3 (0.1, 0.3) | 0.3 (0.2, 0.4) | 0.3 (0.2, 0.3) |  |  |  |  |
| Orexin, pg/mL | A | 75.04 (64.71, 78.76) | 59.41 (57.13, 67.86) | 54.71 (52.92, 66.77) | 57.88 (56.96, 59.65) * | 0.029 | 0.716 | 0.752 | 0.366 |
|  | W | 57.22 (52.37, 73.82) | 63.9 (56.17, 74.60) | 60.80 (52.57, 76.44) | 62.82 (52.64, 73.07) |  |  |  |  |
| Leptin, ng/mL | A | 11.6 (7.6, 20.3) | 4.1 (2.9, 11.9) *** | 6.0 (2.6, 10.9) *** | 4.7 (2.3, 9.9) *** | 0.712 | 0.712 | 0.634 | 0.917 |
|  | W | 13.1 (8.8, 22.9) | 6.6 (3.2, 9.0) *** | 5.5 (3.6, 11.9) *** | 5.0 (3.5, 9.3) *** |  |  |  |  |

Data are means ± SD, or median (25%, 75%). *P < 0.05, **P <0.01, and ***P < 0.001 for comparison with the Week 0 value in the same group. P value for comparison of A group with W group value at the same time point. Abbreviations: LH, luteinizing hormone; FSH, follicular stimulating hormone; FBG, fasting blood glucose; 2h PG, 2h plasma glucose; AUC-Glucose, the glucose area under the curve of OGTT; FINS, fasting plasma insulin; AUC-Insulin, the insulin area under the curve of OGTT; HOMA-IR, Homeostasis model assessment for insulin resistance index; HbA1c, Hemoglobin A1c; BMI, body mass index; TC, total cholesterol; TG, triglyceride; ALT, alanine aminotransferase; AST, aspartate transaminase; GGT, γ-glutamyltransferase; LBP, lipopolysaccharide-binding protein; α-AGP, α-1-acid glycoprotein.

| **TABLE S2 \|** The components of the two ready-to-consume prepared foods of WTP diet. | | | | | |
| --- | --- | --- | --- | --- | --- |
|  | Canned gruel | Prebiotic powder |  | Canned gruel | Prebiotic powder |
| Ash content (g/100g) | 0.45 | 0.067 | Iodine (mg/kg) | 0.12 | 0.05 |
| Moisture (g/100g) | 80.5 | 4.2 | Chlorine (mg/100g) | 32.6 | < 10 |
| Energy (kJ/100mL) | 333 | 1407 | Selenium (mg/kg) | 0.016 | - |
| Carbohydrate (g/100g) | 12.5 | 71.2 | Chromium (mg/kg) | - | - |
| Protein (g/100g) | 3.63 | < 0.1 | Fluorine (mg/kg) | < 0.5 | < 0.50 |
| Fat (g/100g) | 1.2 | < 0.1 | Inositol (mg/kg) | 90 | - |
| Fiber (g/100g) | 1.7 | 24.5 | Linoleic acid (g/100g) | 0.28 | - |
| Soluble fiber (g/100g) | 0.2 | 24.5 | α-linolenic acid (g/100g) | 0.01 | - |
| Insoluble fiber (g/100g) | 1.6 | < 0.1 | Docosahexenoic acid (g/100g) | - | - |
| Vitamin A (mg/kg) | - | - | Eicosatetraenoic acid (g/100g) | - | - |
| Vitamin D (mg/kg) | - | - | Nucleotide (mg/100g) | - | - |
| Vitamin E (mg/kg) | 2.8 | - | Choline (mg/100g) | 26 | 9 |
| Vitamin K1 (μg/100g) | - | - | L-carnitine (mg/kg) | - | - |
| Vitamin B1 (mg/100g) | - | - | Taurine (mg/100g) | 13.8 | - |
| Vitamin B2 (mg/100g) | 0.082 | 0.052 | Molybdenum (mg/kg) | - | - |
| Vitamin B6 (μg/100g) | - | - | Cobalt (mg/kg) | - | - |
| Vitamin B12 (μg/100g) | - | - | Aspartic acid (g/100g) | 0.35 | - |
| Vitamin C (mg/100g) | < 0.3 | < 0.3 | Threonine (g/100g) | 0.13 | - |
| Biotin (μg/100g) | - | - | Serine (g/100g) | 0.19 | - |
| Niacin (μg/100g) | 220 | - | Glutamic acid (g/100g) | 0.7 | - |
| Vitamin B5 (μg/100g) | - | - | Proline (g/100g) | 0.2 | - |
| Folate (μg/100g) | 9.31 | 2.65 | Glycine (g/100g) | 0.16 | 0.01 |
| Sodium (mg/kg) | 67 | 77 | Alanine (g/100g) | 0.22 | - |
| Potassium (mg/kg) | 1798 | 21 | Valine (g/100g) | 0.19 | - |
| copper (mg/kg) | 1 | - | Cystine (g/100g) | 0.1 | - |
| Magnesium (mg/kg) | 337 | 4 | Methionine (g/100g) | 0.06 | - |
| Iron (mg/kg) | 9 | 2 | Isoleucine (g/100g) | 0.16 | - |
| Zinc (mg/kg) | 5 | 2 | Leucine (g/100g) | 0.34 | - |
| Manganese (mg/kg) | 5 | - | Tyrosine (g/100g) | 0.13 | - |
| Calcium (mg/kg) | 158 | 4 | Phenylalanine (g/100g) | 0.2 | - |
| Phosphorus (mg/100g) | 74.2 | < 2 | Histidine (g/100g) | 0.09 | - |
| Iodine (mg/kg) | 0.12 | 0.05 | Trytophan (g/100g) | 0.03 | - |
| Chlorine (mg/100g) | 32.6 | < 10 | Lysine (g/100g) | 0.17 | - |
| Selenium (mg/kg) | 0.016 | - | Arginine (g/100g) | 0.25 | - |
| Chromium (mg/kg) | - | - | Total amino acid (g/100g) | 3.67 | 0.01 |
| Fluorine (mg/kg) | < 0.5 | < 0.50 |  |  |  |

Canned gruel: The concentration of each nutrient is per 100 g wet weight; Prebiotic powder: The concentration of each nutrient is per 100 g dry weight.

**TABLE S3 |** Taxonomical information of the 132 ASVs in 14 CAGs. (separate file)

**References**

1. Zhao L, Zhang F, Ding X, Wu G, Lam YY, Wang X, et al. Gut bacteria selectively promoted by dietary fibers alleviate type 2 diabetes. *Science* (2018)359(6380):1151-1156. doi:10.1126/science.aao5774
